# Seroprevalence and Risk Factors of Crimean-Congo Hemorrhagic Fever in Cattle of Smallholder Farmers in Central Malawi

**DOI:** 10.3390/pathogens10121613

**Published:** 2021-12-10

**Authors:** Marvin Collen Phonera, Martin Chitolongo Simuunza, Henson Kainga, Joseph Ndebe, Mwelwa Chembensofu, Elisha Chatanga, Setiala Kanyanda, Katendi Changula, Walter Muleya, Benjamin Mubemba, Simbarashe Chitanga, Masahiro Kajihara, Hirofumi Sawa, Gilson Njunga, Ayato Takada, Edgar Simulundu

**Affiliations:** 1 Department of Animal Health and Livestock Development, Ministry of Agriculture, Lilongwe 207203, Malawi; gilsonjunga@gmail.com; 2 Department of Disease Control, School of Veterinary Medicine, University of Zambia, Lusaka 10101, Zambia; martin.simuunza@unza.zm (M.C.S.); hkainga@bunda.luanar.mw (H.K.); j.ndebe@yahoo.com (J.N.); kajihara@czc.hokudai.ac.jp (M.K.); h-sawa@czc.hokudai.ac.jp (H.S.); atakada@czc.hokudai.ac.jp (A.T.); 3 Africa Center of Excellence for Infectious Diseases of Humans and Animals, University of Zambia, Lusaka 10101, Zambia; 4 Department of Veterinary Medicine, Lilongwe University of Agriculture and Natural Resources, Lilongwe 207203, Malawi; chatanga@vetmed.hokudai.ac.jp; 5 Department of Para-Clinical Studies, School of Veterinary Medicine, University of Zambia, Lusaka 10101, Zambia; malronc2003@yahoo.co.uk (M.C.); katendi.changula@gmail.com (K.C.); 6 Laboratory of Parasitology, Graduate School of Infectious Diseases, Faculty of Veterinary Medicine, Hokkaido University, Hokkaido 060-8588, Japan; 7 International Training and Education Center for Health, Lilongwe 207203, Malawi; skanyanda@itech-malawi.org; 8 Department of Biomedical Sciences, School of Veterinary Medicine, University of Zambia, Lusaka 10101, Zambia; waltermuleya@gmail.com; 9 Department of Wildlife Sciences, School of Natural Resources, Copperbelt University, Kitwe 50100, Zambia; mubembab85@yahoo.co.uk; 10 Department of Biomedical Sciences, School of Medicine, Copperbelt University, Ndola 50100, Zambia; 11 Department of Paraclinical Studies, School of Veterinary Medicine, University of Namibia, Windhoek 10005, Namibia; schitanga@gmail.com; 12 Department of Biomedical Sciences, School of Health Sciences, University of Zambia, Lusaka 10101, Zambia; 13 School of Life Sciences, College of Agriculture, Engineering and Sciences, University of KwaZulu-Natal, Durban 4000, South Africa; 14 Division of Global Epidemiology, International Institute for Zoonosis Control, Hokkaido University, Sapporo 001-0020, Japan; 15 Division of Molecular Pathobiology, International Institute for Zoonosis Control, Hokkaido University, Sapporo 001-0020, Japan; 16 Division of International Research Promotion, International Institute for Zoonosis Control, Hokkaido University, Sapporo 001-0020, Japan; 17 Global Virus Network, Baltimore, ML 21201, USA; 18 International Collaboration Unit, International Institute for Zoonosis Control, Hokkaido University, Sapporo 001-0020, Japan; 19 One Health Research Center, Hokkaido University, Sapporo 001-0020, Japan; 20 Macha Research Trust, Choma 20100, Zambia

**Keywords:** cattle, Crimean-Congo hemorrhagic fever virus, Malawi, seroprevalence

## Abstract

Crimean-Congo hemorrhagic fever virus (CCHFV) is endemic in Africa, Asia, and Eastern Europe where it circulates among animals and ticks causing sporadic outbreaks in humans. Although CCHF is endemic in sub-Saharan Africa, epidemiological information is lacking in many countries, including Malawi. To assess the risk of CCHF in Malawi, we conducted an epidemiological study in cattle reared by smallholder livestock farmers in central Malawi. A cross-sectional study was conducted in April 2020 involving seven districts, four from Kasungu and three from Lilongwe Agriculture Development Divisions. A structured questionnaire was administered to farmers to obtain demographic, animal management, and ecological risk factors data. Sera were collected from randomly selected cattle and screened for CCHF virus (CCHFV) specific antibodies using a commercial ELISA kit. Ticks were collected from cattle and classified morphologically to species level. An overall CCHFV seropositivity rate of 46.9% (*n* = 416; 95% CI: 42.0–51.8%) was observed. The seropositivity was significantly associated with the age of cattle (*p* < 0.001), sex (*p* < 0.001), presence of ticks in herds (*p* = 0.01), district (*p* = 0.025), and type of grazing lands (*p* = 0.013). Five species of ticks were identified, including *Hyalomma truncatum*, a known vector of CCHFV. Ticks of the species *Hyalomma truncatum* were not detected in two districts with the highest seroprevalence for CCHF and vector competency must be further explored in the study area. To our knowledge, this is the first report of serologic evidence of the presence of CCHV among smallholder cattle in central Malawi. This study emphasizes the need for continued monitoring of CCHFV infection among livestock, ticks, and humans for the development of data-based risk mitigation strategies.

## 1. Introduction

Crimean-Congo hemorrhagic fever (CCHF), caused by the Crimean-Congo hemorrhagic fever virus (CCHFV), is a tick-borne zoonotic disease that may cause severe disease in humans [1]. CCHFV is a member of the order *Bunyavirales*, family *Nairoviridae*, and genus *Orthonairovirus* [2] and is considered one of the widely distributed arbovirus infecting both wild and domestic vertebrates [3]. Serological and molecular studies have provided evidence of CCHFV presence in ticks and clinically healthy non-human mammals and avian species [1,4,5,6,7], suggesting natural circulation in these hosts. When spillover to humans occurs, CCHFV can cause fatal outbreaks [8,9]. The seasonality of tick dynamics in endemic regions appears to correspond to seasonal waves of CCHF episodes in humans [1,10]. Transmission to susceptible humans is commonly by bites of *Hyalomma* ticks, which are known principal vectors of CCHFV [11,12] as well as through direct contact with body fluids and tissues of viremic animals and infected humans [8,11,13].

Whilst most infections in humans are asymptomatic (≈88%), the clinical disease does occur and is characterized by signs that range from mild fever to severe hemorrhagic disease along with multiple organ failure and often result in death. Further, high case fatality rate (10–50%) has been recorded [14,15,16,17]. Although CCHF is of great public health significance, to date, no approved curative chemotherapy nor vaccine is available to mitigate its impact [18,19,20,21,22].

Geographically, about 50 countries across Africa, Asia, and Eastern Europe are considered endemic to CCHFV and *Hyalomma* ticks [21,23]. It is estimated that over three billion people are at risk of infection, with 10,000–15,000 infections annually resulting in about 500 deaths per annum [17]. Through a meta-analysis, the global mean seroprevalence of CCHFV was estimated at 18.6% for cattle alone and 24.7% for all domestic animals [23]. In Africa, the seroprevalence in animals ranges from 0.4 to 75% [4,24]. Its spatial distribution drivers include long-distance live animal trade, habitat fragmentation, expansion of agricultural/cultivation lands, and increase in environmental mean temperatures [17,25]. Migratory birds are also implicated in the spread of CCHFV by carrying infected ticks over long distances [26,27].

CCHFV is considered a serious occupational hazard among people working along the livestock production value chain, which includes farmers, animal handlers, abattoir workers, and veterinarians because of increased exposure to tick bites and viremic animal body fluids [12,13,27,28]. Further, human-to-human transmission occurs commonly in healthcare facilities [4,29,30]. In recent years, there have been increased reports of CCHF amongst travelers (tourists) who are diagnosed with the infection upon return to their respective non-endemic countries [31]. These increased reports in tourists could be associated with engagement in high-risk activities (game trekking) but could be also indicative of the lack of diagnostic and surveillance capacity in these endemic developing countries [32].

There is a lack of epidemiologic information about the presence of CCHF in Malawi despite serologic and/or molecular evidence for its presence in surrounding countries, including Mozambique, Tanzania, Zimbabwe, Namibia, South Africa, Democratic Republic of Congo, Kenya, Uganda, and Zambia [3,4,24,29,33,34]. Malawi’s agriculture sector has changed in various aspects such as tick control strategy (from public-owned to community-owned, in the mid-1990s), increased within and cross border animal movements, and expansion of cultivation and grazing lands into natural forests and marginalized lands following human population growth [35,36,37,38]. Currently, *dambos* (seasonally waterlogged depressions or wetlands) and uplands (elevated and generally dry areas) are the main source of grazing land for livestock. The concurrent existence of the competent vectors (*Hyalomma* ticks) and favorable ecological risk factors [39,40], suggests an increased potential risk of CCHF emergence in Malawi. Hence, this study aimed at providing epidemiological data on the seroprevalence of CCHFV infection and assessing its associated risk factors in cattle in central Malawi.

## 2. Results

### 2.1. Description of the Study Population

**Figure 1:**
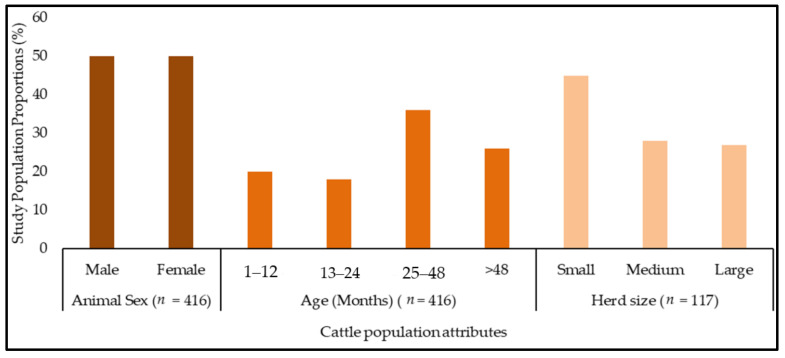
The study cattle population structure by sex, age, and herd size. NB: Herd size categories: small = 1–6 animals, medium = 7–14 animals, and large >14 animals.

A total of 416 cattle, with 208 being male, from 117 cattle herds were sampled. The calculated sample size was 436 cattle (see Section 4). The determined sample size could not be achieved due to poor roads in some veterinary stations. A structured questionnaire was administered to 108 (103 males; 5 females) cattle owners. Figure 1 below shows the study cattle population herd structure. The population had many cattle aged >24 months, and most of them belonged to small herd sizes.

Management of cattle and tick infestation levels varied among the cattle herds (Table 1). About 80.3% (94/117) of cattle herds were grazed in dambo lands. Ticks were present in 90.6% (106/117) of the cattle herds. Tick control was reported to be practiced in 62.0% (67/108) of the herds. The majority of the cattle farmers, 50.9% (55/108), used the spraying method, and only one farmer, 0.9% (1/108), plunge dipped his cattle.

### 2.2. Tick Species Identified on Cattle

**Figure 2:**
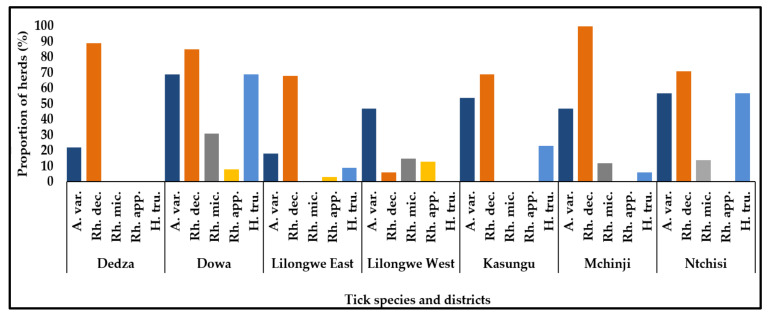
Proportions of cattle herds infested with various tick species in the study districts. Abrevattions: A. var. = *Amblyomma variegetum*; Rh. dec. = *Rhipicephalus decoloratus*; Rh. mic. *= Rhipicephalus microplus*; Rh. App. *= Rhipecephalus appendiculatus*; and H. tru. *= Hyalomma truncatum*.

Five species of ticks were identified from the sampled cattle herds. *Rhipicephalus decoloratus* was present in all the sampled districts with herd infestation ranging from about 10.0% in Lilongwe West to 100% in Mchinji district. *Hyalomma truncatum* was present in 5 (Ntchisi, Dowa, Lilongwe East, Kasungu, and Mchinji) of the 7 study districts. Dowa had the highest herd infestation level of *Hyalomma truncatum* (70.0%) and whereas this species of tick was not observed in Dedza and Lilongwe West districts. *Amblyomma variegetum*, *Rhipicephalus microplus*, and *Rhipecephalus appendiculatus* were also present in the cattle herds. Figure 2 shows the distribution of the ticks in the cattle herds for each study district.

### 2.3. Seroprevalence of CCHFV Infection in Cattle

**Figure 3:**
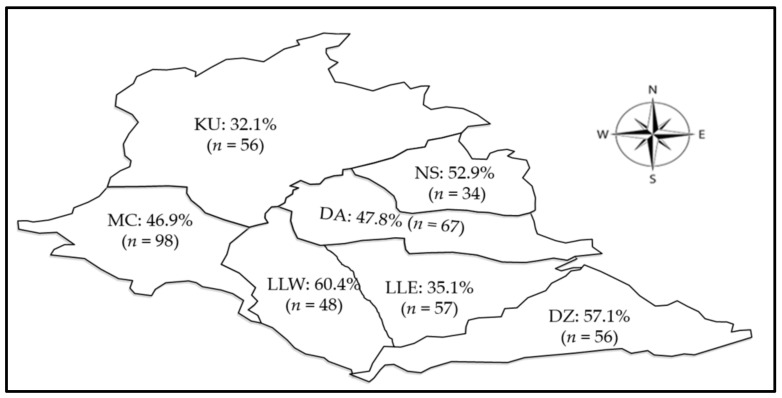
Spatial distribution of seropositivity (%) to CCHFV in cattle in the study area. Abbreviations: DZ = Dedza, DA = Dowa, LLE = Lilongwe East, LLW = Lilongwe West, KU = Kasungu, MC = Mchinji, and NS = Ntchisi.

Individual cattle optic densities data used for the determination of cattle being positive for CCHFV antibody are shown in Figure S1 (Supplementary Materials available online). Out of 416 cattle, CCHFV antibodies were detected in 195 cattle, representing a seroprevalence of 46.9% (95% CI = 42.0–51.8). The seropositivity varied across the study sites (Figure 3), with the highest seroprevalence being observed in Lilongwe West (60.4%; 95% CI = 45.3–74.3%), followed by Dedza (57.1%; 95% CI = 43.2–70.3%) and the least seroprevalence was in Kasungu (32.1%; 95% CI = 20.3–46.0%).

### 2.4. Risk Factors Associated with Detection of CCHFV-Specific Antibodies in Cattle

Bivariate analysis (*p*-value < 0.25 cut-off point) was used to determine which risk factors were significantly associated with CCHF seropositivity (Table 2). These risk factors were district, age, sex, ticks on the herd, grazing land type, animal source, and herd size.

The maximum likelihood estimates for the risk factors that were significantly associated with CCHFV seropositivity in bivariate analysis (Table 3) were estimated. The odds of cattle being seropositive for CCHFV were more than four times for those older than 24 months when compared to those of twelve months and below. In addition, The odds of female cattle being seropositive were more than twice that of male cattle, while those with ticks were more than three times more likely to be seropositive than those that had no ticks. Cattle grazing in uplands were more than four times more likely to be seropositive than those grazing in the dambo. Further, seroprevalence differed significantly among some of the study districts.

## 3. Discussion

As the potential of emerging and re-emerging infectious diseases to cause public health emergencies such as pandemics is on the rise [41], epidemiological data of different pathogenic infectious agents is urgently needed to inform risk mitigation strategies. CCHF is increasingly becoming a global threat with the increased number of human cases being reported in the Middle East and the Balkans peninsula in the past decade [42]. In Africa, apart from South Africa where cases have been reported for decades [3,43], a number of human cases of CCHF were reported recently in Uganda [9] and Namibia [3]. CCHFV has been reported in several African countries in humans, animals, or ticks [4,23,44,45]. However, there are some countries whose CCHFV status is not known, and such countries are considered CCHFV free. The lack of, or poor, surveillance systems has been assumed to account for the failure of CCHFV detection in such countries. Malawi’s health surveillance system has been described as poor [46], with no surveillance system specific for CCHF, but the country is considered to be CCHF free. However, Malawi falls within a high potential risk region for CCHF occurrence because of the presence of *Hyalomma* ticks and conducive tropical climate [39,40,45]. Furthermore, CCHFV has been detected in cattle and ticks in the eastern province of Zambia [34], a region sharing a boundary with the study areas of this report. As such, the country requires close monitoring of CCHFV as well as other emerging and re-emerging vector-borne infectious diseases.

For the first time in Malawi, we report the exposure of cattle to CCHFV in the central region. These results support the idea of CCHFV infections occurring in animals in African countries where no human cases of CCHF have previously been reported. CCHF may be undetected due to lack of diagnostic capacity or lack of knowledge on CCHF among clinicians. Local and cross-border uncontrolled animal movement (through movements of viremic hosts) in Africa, Europe, and the Middle East, has been suggested as one of the mechanisms by which a vector-borne virus closely related to CCHFV, Rift Valley Fever virus is spreading in these regions [47,48,49]. In addition, uncontrolled animal movement due to porous borders in eastern, central, and southern Africa, has contributed to the spreading of different genotypes of the African swine fever virus (ASF) [50,51]. Similarly, this may also explain the spread of other infectious agents such as CCHFV within the region. The cattle seroprevalence reported in this study (46.9%) is comparatively higher than the global mean cattle CCHFV seroprevalence of 18.6% [23]. It was also high when compared to CCHFV seroprevalence reported in the Democratic Republic of Congo (DRC) (0.4%) [4,52]. However, it is less than what has been reported in Uganda (75.0%), Mali (66.0%), Mauritania (67.0%), and Senegal (57.1%) [24,53,54,55].

Apart from the true variation in seroprevalence, the reported seroprevalence rates are also dependent on the diagnostic tests used [4,24]. For instance, the CCHFV double antigen ELISA test employed in the present study detects both IgG and IgM [56] and uses a larger volume of serum sample [57], compared to other forms of ELISA tests that detect either IgG or IgM only, a scenario which may contribute higher seroprevalence. However, some studies which have used an ELISA method that detects only IgG or IgM CCHFV antibodies have reported higher seroprevalence than those of the present study [4,58,59], indicating that other factors could be at play.

The seroprevalence of CCHFV is associated with many risk factors. Geographical location, sex, age, and presence of ticks in cattle herds have been reported and discussed in previous studies as among the risk factors for CCHFV [55,59,60,61]. In addition, this study found high seroprevalence in cattle grazed in uplands compared to those grazed in dambos. *Hyalomma* ticks prefer drier environments [62], making uplands more likely to have CCHFV vector ticks compared to the dambos, which are wet most of the time. However, in this study, high seroprevalence was observed in Lilongwe West and Dedza, districts that had no *Hyalomma* ticks. Since tick activity varies with the season of the year [62] and in the present study samples were collected at a single point in time, the generated results on prevailing ticks are not enough evidence to conclude the absence of *Hyalomma* ticks and other tick species in other districts. Further, CCHFV had been detected in many other tick genera as *Rhipicephalus*, *Amblyomma*, though, vector competency has not been confirmed in these ticks [1,63,64,65]. Based on these observations, vector competency of tick species other than *Hyalomma* species has to be explored in this study area.

Some studies have reported no association between the sex of cattle [60] and of camels [61] and being CCHFV seropositive. However, one study reported that cattle gender was associated with the risk of an animal being infected with CCHFV [55], a finding which is similar to the results of this present study where female cattle were observed to have a higher risk than males. Female cattle are raised mainly for breeding purposes making them spend more time in the fields grazing and thus have an increased risk of being exposed to ticks. In contrast, older male cattle are used for drought power and stay away from grazing areas longer than female and young cattle, particularly during the rainy season when cultivation of crops is at its peak. This period also coincides with increased tick activity. Consequently, we surmise that male cattle may be less frequently exposed to ticks and this could explain why female animals are at increased risk of CCHFV exposure.

Older cattle had a higher odds of being CCHFV seropositive compared to younger ones (12 months or less). Cumulative exposure to ticks and tick-borne pathogens increases as animals age [66], thus explaining the higher odds of CCHFV seropositivity in older animals. The presence of other livestock species was not found to be a risk factor for CCHFV seropositivity in the present study. This is similar to the findings of Adam et al. [60], who also observed no significant association between the presence of other livestock and increased CCHFV seropositivity. In the current study, a larger proportion (99.1%) of farmers also kept other livestock species. Thus, there was not enough representation of farmers keeping cattle only to generate enough statistical power to detect such a difference if it existed in our study population.

This study found that the absence of ticks on cattle was associated with reduced odds of cattle being seropositive for CCHFV. A reduced CCHFV seropositivity was also observed to be associated with the absence of ticks in camels (*Camelus dromedaries*) [61]. However, the odds ratio was not statistically significant between cattle where ticks were controlled and those in which ticks were not controlled, an observation that has been reported previously [60]. Cattle raised communally mingle during grazing with other cattle herds and other livestock in general. Such communal cattle grazing along with irregular tick control protocols in some cattle herds can render tick control efforts ineffective.

This study is not without limitations. A limitation in this study includes the use of a questionnaire to obtain information regarding farmer demographics and the management of animals. This approach is subject to recall liabilities and truthfulness of the respondents. The study was also limited by a lack of supportive information like information on tick resistance to acaricides, in the study area, which could also help to explain or justify the ineffectiveness of tick control measures.

## 4. Materials and Methods

### 4.1. Study Sites

**Figure 4:**
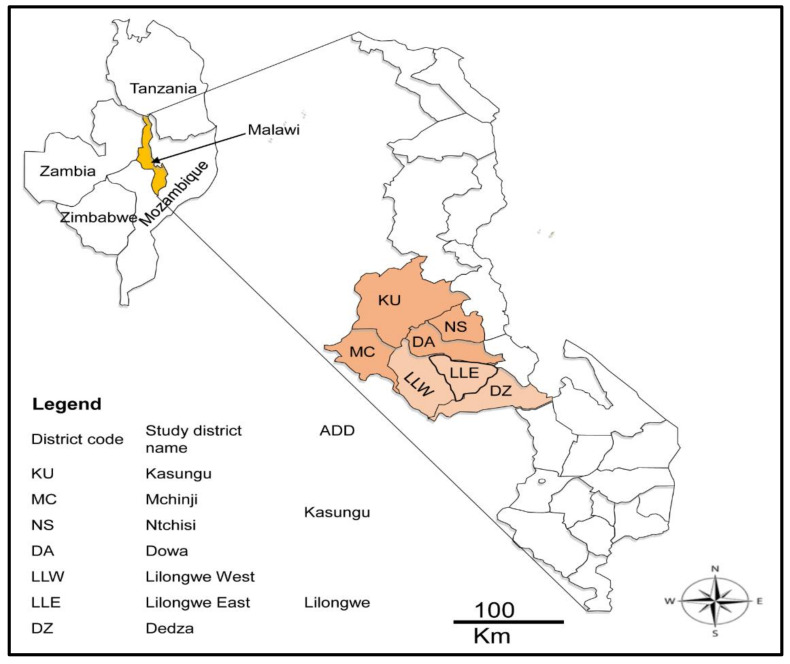
Map of Malawi showing the seven study districts and Malawi’s neighboring countries.

The study was conducted in the central region of Malawi in April 2020. Malawi, located in southern Africa, is a landlocked and agriculture-based country covering 118,484 km^2^. It is located within latitudes 9° and 18°S, and longitudes 32° to 36° E and is bordered by Tanzania to the north, Mozambique to the east, south, and southwest, and Zambia to the west. The study was conducted in the Lilongwe-Kasungu plain (covering Kasungu and part of Lilongwe agricultural development divisions (ADD) in the central part of the country (Figure 4). The plain has a savanna tropical climate and experiences a hot-dry summer (September to November), hot-wet summer (December to April; rainfall ranges from 750 to 1200 mm per annum), and moderate winter (May to August) seasons. The study area for the present study, Lilongwe-Kasungu plain, is known to be infested with *Hyalomma* ticks [37] and was thus purposefully selected.

### 4.2. Study and Sampling Design

The study was cross-sectional in design. Sample collection centers (village centers or veterinary stations) were randomly identified in the study districts. A herd was defined as all cattle groupings under one management custody [67]. Smallholder cattle farmer registers were used as sampling frames. As such, herds were selected using a systematic random sampling technique. Since individual cattle were not identified within herds, arbitrary numbers were assigned to individual animals within a herd which was later used for simple random selection by a raffle draw. The study included cattle of all ages and sexes that were raised communally in the study area. However, the study excluded heavily pregnant (second and third trimesters) cows and clinically ill cattle to avoid stressing the animals.

The sample size was determined through proportional probability using Ausvet EpiTools software (http://epitools.ausvet.com.au/ accessed on 6 May 2019). A total sample size of 436 cattle was determined using the following parameters: 50% prevalence (no established prevalence in the study area was available), 6% relative precision, 95% confidence level, and 1.5 design effect [68].

### 4.3. Questionnaire Administration

A structured questionnaire, in the local language (*Chewa*), was administered to cattle farmers through face-to-face interviews. The questionnaire was designed to collect information such as smallholder farmer demographics, herd size categorized as small (1–6 animals), medium (7–14 animals), and large (>14), source of animals (within or outside the district); ticks on herd (present or absent), tick control measures (spraying or dipping), and type of grazing land (dambo = low lying waterlogged wetlands; upland = elevated and generally drier; or in both (dambo and upland)). Selected cattle owners who did not consent to participate in the questionnaire or to allow their animals to be sampled were replaced by other cattle owners from the same sample collection center.

### 4.4. Cattle Attributes

Due to lack of written records, cattle owners recalled the age of the sampled animals, and when in doubt, the investigator estimated the age by dentition method [69,70]. Animal sex and the presence of ticks were recorded as male or female and present or absent, respectively, following a visual inspection.

### 4.5. Sample (Sera and Tick) Collection from Cattle, Storage, and Transportation

Approximately five (5) milliliters of whole blood was aseptically collected in a plain vacutainer tube from each sampled animal through the jugular or coccygeal venipuncture approaches. Iodine, to clean the blood collection site, and sterile disposable needles were used to achieve aseptic standards. Sample tube labeling included animal number, district, collection center, herd number, date of sample collection, and sample type. Whole blood samples were allowed to clot overnight before serum was separated by centrifugation at 1000× *g* for 15 min as per World Organization for Animal Health protocol [71] and later aliquoted into two milliliter Eppendorf tubes.

Ticks were handpicked from cattle body surfaces. The picked ticks were collected in 50 mL falcon tubes with perforated lids for ventilation. Fresh pieces of grass/leaves were added to each tube to provide humidity for the ticks. Each tube was labeled according to the district, collection center, herd number, and date of tick collection. Ticks were transported to the Central Veterinary Laboratory (CVL) in Lilongwe, where they were stored at 18 °C until identification using morphological features [62]. Thereafter, both sera and ticks were then stored at −80 °C at the African Union Centre of Excellence for Tick and Tick-borne diseases (AU-CTTBD), Lilongwe, Malawi.

### 4.6. Enzyme-Linked Immunosorbent Assay (ELISA)

All the 416 serum samples were subjected to sandwich ID Screen^©^ CCHF Double Antigen Multi-Species Enzyme-Linked Immunosorbent Assay test (IDvet, Grabels, France). All the reagents and controls were provided in the kit and were reconstituted and tests were carried out following the manufacturer’s instructions. All samples and controls were run in duplicates, and the average of the duplicates was considered as the test result. This assay, simultaneously and indiscriminately, detected both IgM and IgG with the sensitivity of 98.9% and specificity of 100% [56]. A test run was considered valid if the mean optic density of the positive control (ODpc) was greater than 0.35, and the ratio of the mean ODpc to mean optic density for negative control (ODnc) was greater than 3. Interpretations of the test ODs were based on the ratio of the mean sample optic density to ODpc, expressed as a percentage (S/p × 100). Samples with S/p% less or equal to 30% were considered negative, and samples with S/p% greater than 30% were considered positive. Table S1 shows validation data for all (10) plates that were run and all the runs were valid on both criteria.

### 4.7. Data Analysis

All data were entered, cleaned, and validated in Microsoft™ excel spreadsheet. The CCHFV ELISA test results (positive or negative) were the only dependent variable in this study. Descriptive and inferential analyses were performed in IBM SPSS version 20 (IBM Corp, Armonk, NY, USA) and MS Office Excel^®^ 2016. Bivariate analysis was performed using the Pearson Chi-Square test of association (and Fisher’s exact test, where appropriate) at a significance level of *p* < 0.25. All the factors that were significant at bivariate analysis were used to model the odds ratios of CCHFV seropositivity. Multivariate analysis was done using a stepwise binary logistic regression model for categorical outcome at the significance level of *p* ≤ 0.05. All the tests were performed at a 95% confidence level. Missing data were coded as −99 and were non-informative in all the models. A significant Omnibus Test for Model coefficients (*p* < 0.050) and a non-significant Hosmer and Lemeshow Test (*p* > 0.050) were used to check whether the model fitted the data.

## 5. Conclusions

This study, for the first time, has provided serologic evidence of the circulation of CCHFV in cattle kept by smallholder farmers in central Malawi and identified several risk factors for CCHFV seropositivity. The study stresses the need for continued monitoring of CCHFV infection among livestock, ticks, and humans to assist with the development of evidence-based control strategies. Countrywide studies to identify potential CCHFV hot spots in animals, vectors and humans are highly recommended for prudent risk mitigation.

## Figures and Tables

**Table 1:** Distribution of cattle herds across different cattle management factors.

Factor (*n*)	Category	Number of Herds per Category	Percentage (95% CI)
Grazing land type (*n* =117)	Dambo	94	80.3 (72.0–87.1)
	Both (dambo and upland)	12	10.3 (05.1–17.2)
	Upland	11	9.4 (4.79–16.20)
Ticks on herd (*n* = 117)	Present	106	90.6 (83.8–95.2)
	Absent	11	9.4 (4.8–16.2)
Tick control (*n* = 108)	Done	67	62.0 (52.2–71.2)
	Not done	41	38.0 (28.8–47.8)
Method of tick control (*n* = 108)	No tick control	41	38.0 (28.8–47.8)
	Spraying	55	50.9 (41.1–60.7)
	Dipping	1	0.9 (0.0–5.1)
	Mixed methods	11	10.2 (5.2–17.5)
Tick control frequency (*n* = 108)	None	41	38.0 (28.8–47.1)
	Whenever necessary	33	30.6 (22.2–40.2)
	Monthly	19	17.6 (10.9–26.1)
	Fortnightly	11	10.2 (5.2–17.5)
	Weekly	4	3.7 (1.0–9.2)
Farmer keeping other stock species (*n* = 108)	Yes	107	99.1 (95.0–100.0)
	No	1	0.9 (0.0–5.1)

*n* = number of herds included per factor; CI = Confidence Interval.

**Table 2:** Summary of test of association analysis between potential risk factors and CCHFV seropositivity.

Risk Factor	Category	*n*	Seroprevalence (%)	95% CI	*p*-Value
District	Dedza	56	57.1	43.2–70.3	0.025 *
	Dowa	67	47.8	35.4–60.3	
	Kasungu	56	32.1	20.3–46.0	
	Lilongwe East	57	35.1	22.9–48.9	
	Lilongwe West	48	60.4	45.3–74.2	
	Mchinji	98	46.9	36.9–57.3	
	Ntchisi	34	59.9	35.1–70.2	
Sex	Male	208	36.5	30.0–43.5	<0.001 *
	Female	208	57.2	50.2–64.0	
Age (Months)	1–12	83	25.3	16.4–36.0	<0.001 *
	13–24	80	31.3	21.4–42.6	
	25–48	151	58.3	50.0–66.2	
	>48	102	59.8	49.6–69.4	
Ticks on herd	Present	384	48.7	43.6–53.8	0.016 *
	Absent	32	25.0	11.5–43.4	
Grazing land type	Dambo	326	44.8	39.3–50.4	0.013 *
	Both (Dambo and upland)	40	40.0	24.9–56.7	
	Upland	50	33.0	51.2–78.8	
Tick control	Done	254	46.9	40.6–53.2	0.854
	Not done	133	45.9	37.2–54.7	
Animal source	Within district	331	57.7	42.2–53.3	0.241 *
	Outside district	56	39.3	26.5–53.3	
Presence of other stocks in herd	Present	383	46.7	41.7–51.9	0.336
	Absent	4	25.0	0.1–80.6	
Herd size	Small	127	52.0	42.9–60.9	0.210 *
	Medium	113	48.7	39.2–58.3	
	Large	176	42.1	34.7–49.7	

*n*= number of cattle involved, CI = confidence interval, * = statistically significant difference at *p*-value ≤ 0.25.

**Table 3:** Summary of maximum likelihood estimates for CCHFV seropositivity by risk factors determined.

Risk Factor	Category	OR	CI	*p*-Value
District	Mchinji	r		
	Dedza	2.2	1.0–4.9	0.050 *
	Dowa	0.6	0.3–1.5	0.309
	Kasungu	0.7	0.3–1.6	0.408
	Lilongwe East	1.2	0.5–2.6	0.669
	Lilongwe West	2.8	1.2–6.5	0.016 *
	Ntchisi	5.1	1.4–18.6	0.013 *
Age (Months)	1–12	r		
	13–24	1.2	0.6–2.6	0.626
	25–48	4.4	2.2–8.6	<0.001 *
	>48	4.3	2.1–9.0	<0.001 *
Animal Sex	Male	r		
	Female	2.5	1.6–4.0	<0.001 *
Ticks on herd	Absent	r		
	Present	3.2	1.2–8.5	0.02 *
Grazing land type	Dambo	r		
	Both (Dambo and Upland)	0.5	0.2–1.5	0.244
	Upland	4.4	1.8–10.9	0.001 *

* Statistically significant difference at *p* ≤ 0.05, OR = Odds Ratio, CI = confidence interval, and r = reference category.

## Data Availability

All data have been provided in the article and/or as Supplementary Materials.

## Supplementary Materials

The following are available online at https://www.mdpi.com/article/10.3390/pathogens10121613/s1, Table S1: mean Optic Densities (OD) for positive and negative controls and their calculated ratios; and Figure S1: Ratios of Optic densities for the sample to Optic density for the positive control in percentages.
