# Supplementary material for: Seroprevalence and Risk Factors of Crimean-Congo Hemorrhagic Fever in Cattle of Smallholder Farmers in Central Malawi
Source: Pathogens. 2021 Dec 10;10(12):1613. doi: 10.3390/pathogens10121613 (PMC8709441; doi:10.3390/pathogens10121613)
Supplement: Supplementary file 1 [file pathogens-10-01613-s001.zip › Figure S1_Ratios of Optic densities for sample to Optic density for the positive control in percentages.pdf]

pathogens

MDPI

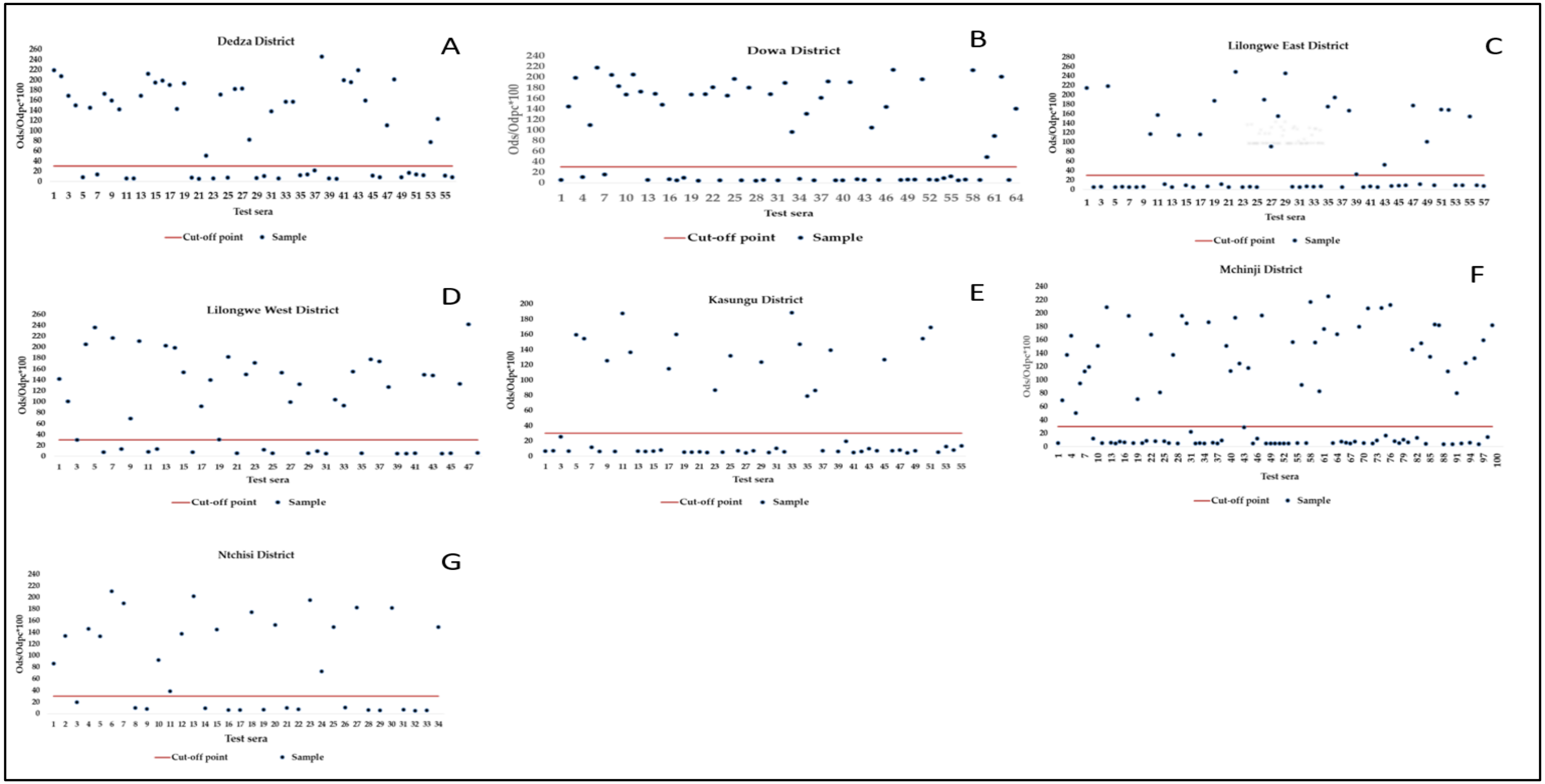

Figure S1: Ratios of Optic densities (OD) for sample (S) to Optic density for the positive control (PC) in percentages used to determine positivity/ negativity of a serum sample (S/P*100). A cut-off point of 30% is shown with a red horizontal line. All study districts individual sera data are shown in charts A-G.
