# Supplementary material for: Seroprevalence and Risk Factors of Crimean-Congo Hemorrhagic Fever in Cattle of Smallholder Farmers in Central Malawi
Source: Pathogens. 2021 Dec 10;10(12):1613. doi: 10.3390/pathogens10121613 (PMC8709441; doi:10.3390/pathogens10121613)
Supplement: Supplementary file 1 [file pathogens-10-01613-s001.zip › Table S1 Mean optic densities for positive and negative controls and their calculated ratios.pdf]

pathogens MDPI

Table S1: Mean optic densities for positive and negative controls and their calculated ratios

Plate ID	A	B	C	D	E	F	G	H	I	J
Mean OD_{pc}	0.799	0.924	0.999	0.888	0.656	1.029	0.888	1.006	0.908	1.456
Mean OD_{nc}	0.046	0.052	0.045	0.046	0.052	0.0485	0.048	0.047	0.056	0.047
OD_{pc}/OD_{nc} ratio	17.549	17.930	22.200	19.516	12.615	21.206	18.489	21.666	16.360	30.968

NB: Mean ODpc were valid when > 0.350 and all ODpc/ODnc were valid when >3.00
OD = optic density; pc = Positive control; nc = Negative control
